# Akinra restores the defective IL-18 NK cell axis in steroid naïve systemic onset JIA patients

**DOI:** 10.1186/1546-0096-9-S1-O33

**Published:** 2011-09-14

**Authors:** W de Jager, SJ Vastert, BJ Noordman, D Holzinger, W Kuis, BJ Prakken, NM Wulffraat

**Affiliations:** 1 Pediatric immunology, University Medical Center Utrecht, Utrecht, Netherland; 2 Institute of Immunology, University Hospital Muenster, Muenster, Germany

## 

Systemic Onset Juvenile Idiopathic Arthritis (SoJIA) is characterized by systemic inflammation and chronic arthritis. Intriguingly, the IL-18-NK cell axis seems to be disturbed in the majority of SoJIA patients. The observed NK cell dysfunction in SoJIA patients contributes to important features of the disease including the susceptibility for macrophage activation syndrome. Here we describe the effects of Anakinra mono treatment on the IL-18-NK cel axis in steroid naïve SoJIA patients. In this study sixteen consecutive patients diagnosed with systemic onset JIA were included. Clinical response to Anakinra was evaluated using the validated core set parameters for JIA as well as several biochemical parameters of disease activity.

In this cohort we show a good clinical response to Anakinra in 14/16 patients SoJIA patients prior to standard steroid treatment. After 3 weeks of treatment 75% of patients achieved a pACR90 score. Clinical improvement was accompanied by normalization of IL-1, IL-6 and IL-18 levels in plasma. Interestingly, the use of Anakinra in patients with short disease duration induces restoration of the IL-18 - NK cell axis resulting in improved lytic NK cell function and regaining of the NK cell responsiveness to IL-18 stimulation. Moreover, Anakinra seems to down regulate inflammasome activation. These data suggest that the mechanisms of inflammatory control induced by Anakinra in SoJIA patients involves more than blocking IL-1R signaling, since it seems to restore the IL-18-NK cell route as well.

